# Lambs supplemented with Amazonian oilseed co-products: Meat quality and fatty acid profile

**DOI:** 10.1371/journal.pone.0293897

**Published:** 2023-12-19

**Authors:** Vinícius Costa Gomes de Castro, Juliana Cristina de Castro Budel, Thomaz Cyro Guimarães de Carvalho Rodrigues, Bruna Almeida Silva, Waléria Cristina Lopes Joset, Alyne Cristina Sodré de Lima, Shirley Motta Souza, Rui José Branquinho Bessa, Suzana Paula Almeida Alves, Jamile Andrea Rodrigues da Silva, Maria Regina Sarkis Peixoto Joele, André Guimarães Maciel e Silva, José de Brito Lourenço-Júnior

**Affiliations:** 1 Institute of Veterinary Medicine, Federal University of Pará, Castanhal, Pará, Brazil; 2 Department of Food Technology, State University of Pará, Marabá, Pará, Brazil; 3 Federal Institute of Amapá, Amapá, Brazil; 4 Federal Institute of the South of Minas Gerais, Machado, Minas Gerais, Brazil; 5 Faculty of Veterinary Medicine, CIISA, Center for Interdisciplinary Research in Animal Health, University of Lisbon, Lisboa, Portugal; 6 Institute of Animal Health and Production, Federal Rural University of the Amazon, Belém, Pará, Brazil; 7 Federal Institute of Pará, Castanhal, Pará, Brazil; University of Illinois, UNITED STATES

## Abstract

The Amazon has a wide variety of oilseeds that generate a huge amount of co-products with potential for use in animal nutrition. The objective was to use alternative resources (oilseed cakes) in the feeding of lambs to assign a sustainable destination to this biomass, and evaluate its influence on the quality and fatty acid (FA) profile of the meat. Twenty-four lambs, male, castrated, crossbred Dorper × Santa Inês, weighing 30 ± 1.3 kg of initial body weight, were distributed in a completely randomized design in 4 treatments (diets) with six replications (animals). The control diet (Control) contained corn and soybean meal as main ingredients, which were partially replaced in the other diets by cupuassu cake diet (Cup), palm kernel cake diet (Palm) and tucuma cake diet (Tuc). The inclusion of Amazon cakes influences the lipid (P = 0.02) and protein (P < 0.01) composition of meat (*longissimus lumborum*); reduces cooking losses (P < 0.01); influences the colors (L, a, b), chroma, and Hue Angle (P < 0.01); promotes changes in total FA composition and FA profile (P < 0.05); reduces hypocholesterolemic FA (h) (P = 0.01), but does not influence hypercholesterolemic (H) and indices h:H, AI and TI (P > 0.05). The inclusion of oilseed cakes influences the chemical composition, physical parameters, composition and fatty acid profile of the meat, but does not influence the indicators of atherogenicity, thrombogenicity and cholesterolemia.

## 1. Introduction

Brazil produces a wide range of oilseeds used, among others, by the food, cosmetic and pharmaceutical industries. The Amazon region stands out in this scenario, being the largest producer of some of these plants: Cupuassu (*Theobroma grandiflorum Schum*.); Oil palm (*Guinean Elaeis Jacq*.); and Tucuma (*Astrocaryum vulgare Mart*.) [1–3]. However, after extracting the raw material, more than 2 billion tons of co-products are generated in the world and are a challenge for the industry, which has encouraged research so that this biomass can be used and the agroindustry advances towards models of circular economy and sustainable production [4,5].

These co-products are usually a good source of protein and energy, but with variable lipid and fatty acid content depending on the oil extraction technology and plant species [6,7]. Due to these characteristics, inclusion in diets for ruminants is an alternative to conventional cereals (corn and soybeans), reducing feed costs and improper disposal of this material [8,9]. However, this inclusion must also consider its lipid profile and secondary metabolism, since meat may be more susceptible to oxidative deterioration (potential negative impact on nutritional properties), shelf life, food quality and general consumer acceptance [10].

The nutritional quality of ruminant meat is mainly determined by its lipid content and fatty acid (FA) composition, as it is the most variable component of meat [11,12]. The improvement in the quality of ruminant meat includes a decrease in saturated fatty acids (SFA) and an increase in monounsaturated and polyunsaturated cis fatty acids (cis-MUFA—PUFA), vaccenic acid (18:1t11), conjugated isomers of linoleic acid (CLA), such as rumenic acid (18:2c9,t11), and decreasing the n-6 PUFA/n-3 PUFA ratio [11,13]. It is noteworthy that 18:2c9,t11 and 18:1t11 are beneficial to consumer health, as they have demonstrated anticarcinogenic activity, and the products from ruminants being the main natural sources of these FAs [11,14].

Cupuassu seed is rich in oleic (18:1c9) and stearic (18:0) acids; and palm kernel and tucuma cakes rich in lauric (12:0) and myristic (14:0) acids, modulators of biohydrogenation [15–17]. However, these saturated FAs have been widely investigated for possibly increasing low-density lipoprotein (LDL) levels, which are related to the onset of cardiovascular disease [18,19]. Thus, the inclusion of these co-products in the diet of lambs and their influence on the final product must be evaluated.

Our hypothesis is that the inclusion of oilseed cakes from the Amazon can influence the quality and fatty acid profile of lamb meat. The objective of the study was to investigate the effects of including these co-products on the criteria of meat quality, profile fatty acids and lipid fraction indices in the meat of feedlot lambs.

## 2. Material and methods

The research was approved by the Ethics Committee for the Use of Animals (CEUA) of the Federal University of Pará - protocol 8694141217.

### 2.1 Animals, diets and treatments

Twenty-four male lambs, castrated (at 60 days of age), crossbred (F1) Dorper × Santa Inês, with 30.0 ± 1.3 kg of initial body weight and age ranging from 4 to 5 months, were used in a completely randomized design, in four treatments (diets). The animals were housed in individual pens (1.5 m^2^), made of wood and suspended, equipped with a feeder, drinker and salt shaker.

The control diet (Control) contained ground corn and soybean meal as main ingredients, which were partially replaced by cupuassu cake diet (Cup), palm kernel cake diet (Palm) and tucuma cake diet (Tuc). The three diets with co-products from the Amazon agroindustry were formulated to be isoenergetic, in order to evaluate the potency of the co-products in substitution of the control treatment. The co-products were purchased from a commercial company, generated from mechanical pressing to obtain the oil. The four diets included 400 g/kg dry matter (DM) of corn silage as a forage component, and 600 g/kg DM of concentrated component, which were homogenized and offered as a total diet. The animals were fed ad libitum, twice a day (7:30 am and 4:30 pm), for 60 days. Samples of ingredients and offered diets were pre-dried in an oven for analysis of the FA profile. The proportion of ingredients, chemical composition of the experimental diets and FA profile are presented in Tables 1 and 2.

**Table 1:** Proportion of ingredients, chemical composition of experimental diets and total dry matter intake.

Items	Diets			
	Control	Cup	Palm	Tuc
**Ingredients (g/kg DM)**				
Corn silage	400	400	400	400
Ground corn	432	62	260	132
Soybean meal	148	68	144	139
Cupuassu seed meal	-	450	-	-
Palm kernel meal	-	-	176	-
Tucuma kernel meal	-	-	-	309
Mineral-vitamin premix^1^	15	15	15	15
Limestone	5	5	5	5
**Chemical composition (g/kg DM)**				
Dry matter	686	687	690	687
Ash	47.9	55.1	51.0	47.1
Crude protein	142	144	142	146
Ether extract	63.8	66.2	66.9	69.9
NDFcp^2^	308	384	385	445
ADFcp^3^	138	193	215	256
Lignin	132	107	100	77
Total digestible nutrients	691	680	679	679
Total dry matter intake (kg)	71.61	75.70	64.28	51.53

Diets: Control = No addition of Amazon oilseed cakes; Cup = inclusion of cupuassu cake; Palm = inclusion of palmiste cake; Tuc = inclusion of tucuma cake. NDF: Neutral detergent fiber corrected for ashes and protein; ADF: Acid detergent fiber corrected for ashes and protein.

^1^Calcium 140 g. Phosphorus 65 g. Magnesium 10 g. Sulfur 12 g. Sodium 130 g. Cobalt 80 mg. Iron 1.000 mg. Iodine 60 mg. Manganese 3.000 mg. Selenium 10 mg. Zinc 5.000 mg. Fluor (max) 650 mg. Vitamin A 50.000 IU. Vitamin E 312 IU

^2^ Neutral detergent fiber corrected for ash and protein

^3^ Acid detergent fiber corrected for ash and protein.

**Table 2:** Fatty acid profile of experimental diets.

Fatty acid, mg/g meat	Diets			
	Control	Cup	Palm	Tuc
C8:0	0.00	0.00	0.10	0.66
C10:0	0.00	0.29	0.14	1.33
C12:0	0.00	0.26	1.80	53.84
C14:0	0.00	0.16	0.83	25.05
C16:0	3.26	7.29	2.12	11.80
C17:0	0.05	0.10	0.08	0.04
C18:0	0.74	17.23	0.93	5.69
C18:1c9	5.03	25.44	2.86	23.48
C18:1c11	0.17	0.47	0.00	0.39
C18:2n-6	6.15	9.02	1.00	9.36
C20:0	0.16	5.44	0.07	0.95
C18:3n-3	0.29	0.66	0.15	0.86
C22:0	0.09	1.00	0.09	0.22
C24:0	0.14	0.21	0.06	0.18

Diets: Control = No addition of Amazon oilseed cakes; Cup = inclusion of cupuassu cake; Palm = inclusion of palmiste cake; Tuc = inclusion of tucuma cake.

### 2.2 Slaughter and meat samples

At the time of slaughter, the animals were stunned by electronarcosis, followed by bleeding, skinning and evisceration, respecting the procedures for handling and humane slaughter of animals [20]. Soon after, the carcasses were kept in a cold room (6°C) for 24 hours. From the left half of the carcass, *Longissimus lumborum* was collected, vacuum packed, taken to the laboratory and frozen at –18°C for physicochemical and fatty acid analysis. For analysis of FA, approximately 50 g (wet weight) of the muscle (per animal) were collected and frozen at -80°C until lyophilization. Samples were lyophilized to constant weight using a Christ Alpha 1–2 LDplus lyophilizer (Christ alpha, Osterode am Harz, Germany).

### 2.3 Meat composition and quality measures

Moisture was analyzed by drying the sample in an oven at 65°C and subsequently at 105°C until constant weight, and the ash was obtained by heating the sample in a muffle at 550°C. Total N was determined by the Kjeldahl method and CP 6.25 × N, according to the standard protocol. The intramuscular fat content of meat was analyzed using the Goldfish method [21], according to the equation: % Fat = DF—DFW/PA x 100, where DF = empty distillation flask weight, DFW = distillation flask weight + ether extract and PA = weight of the collected sample.

Meat pH was determined with a digital pH meter (24 hours after slaughter, in the *Longissimus lumborum* muscle) and water activity with an Aqualab (model 4TE duo). Meat color was determined 24 hours after slaughter, after 30 minutes of exposure of the sample to air, with a Konica Minolta CR-410 Chromometer using the CIE system L* (brightness), a* (red), b* (yellow), ranging from 0 (black) to 100 (white), red (+a) to green (-a), and yellow (+b) to blue (-b). Color saturation (Chroma, C*) and Hue angle (H°) were calculated as C* = (a*2 + b*2)1/2 and h° = tan-1 (b/a), using variables by heart.

The water-holding capacity (WHC) was performed in triplicate, in two meat plates (5 g). The resulting meat sample was weighed and, by difference, the amount of water lost was calculated [22]. To measure cooking losses, three samples (3.0 x 4.0 x 2.5 cm) from each treatment were weighed and cooked to 70°C (internal), measured with a thermocouple. They were then cooled to room temperature and weighed again. The loss is the result of the difference between the initial and final weight of the samples [23].

For shear force (tenderness meat), the texture analyzer TA.XT Plus Stable Micro Systems was used, coupled to a Warner-Bratzler blade, with a descent velocity of 5.0 mm/s. Samples were prepared by removing at least six meat cylinders measuring 1.27 cm in diameter from each steak, with muscle fibers oriented longitudinally. The samples, were then, subjected to shear force (by the blade), and the force in the first cut was recorded and expressed in N/cm^2^ [24].

### 2.4 Fatty acid analysis

The FA from the experimental diets and from the muscle of the lambs were analyzed by gas chromatography (GC) with FA methyl ester (FAME) derivatives. The preparation of FAME from the experimental diets was performed using a transesterification procedure adapted from Palmquist and Jenkins [25]. About 200 mg of feed samples were allowed to react with 1.25 M hydrogen chloride in methanol for 2 h at 70°C with toluene and 1 mL of methyl nonadecanoate (1 mg/mL) as an internal standard.

FAME from muscle and fat samples (previously lyophilized and homogenized) were prepared by a sequential direct transesterification procedure [26]. About 200 mg of muscle was allowed to react with a 0.5 N solution of sodium methoxide in methanol at 50°C, followed by reaction with a 1.25 M solution of hydrogen chloride in methanol at 80°C. The FA methyl esters were extracted with hexane and 1 mg of methyl nonadecanoate (internal standard) was added before transesterification.

Fatty acid methyl esters were analyzed by flame ionization detection GC (GC-FID) using a Shimadzu 2010-Plus (Shimadzu, Kyoto, Japan) equipped with an SP-2560 (100 m × 0.25 mm × 0 .20 μm, Supelco, Bellefonte, PA, USA) capillary column. The chromatographic conditions were: injector and detector temperature maintained at 220°C and 250°C, respectively; Initial oven temperature (50°C) maintained for 1 min, increased by 50°C/min to 150°C and held for 20 min, increased by 1°C/min to 190°C and finally increased by 2°C/ min, until reaching 220°C and maintained at this temperature for 30 min.

Helium was used as carrier gas at a flow rate of 1 mL/min. Samples were injected (1–2 mg FAME/mL) with a split ratio of 1:50 and 1:70, for food and muscle, respectively. FAs were identified by comparing their retention times with the methylated FA standard (FAME). Additional identification of FAME was obtained by mass spectrometry using a Shimadzu GC-MS QP2010 Plus (Shimadzu, Kyoto, Japan).

The GC conditions used in the GC-MS were similar to the GC-FID and MS conditions were as follows: ion source temperature, 200°C; interface temperature, 220°C; ionization energy, 70 eV; scan, 50–500 atomic mass units [26]. Stearoyl-CoA desaturase (SCD) activity was estimated by the ratio between each pair of substrate and product [27].

To assess the nutritional quality of the lipid fraction, the atherogenicity (AI) and thrombogenicity (TI) indices were calculed [28]. In addition, the concentrations of hypercholesterolemic and hypocholesterolemic fatty acids and their index = hypocholesterolemic:hypercholesterolemic (h:H) were evaluated [29]. All calculations were estimated using the following equations:

- Atherogenicity = (C12:0) + C14:0 x 4 + C16:0) / (MUFA + ω6PUFA + ω3PUFA);
- Thrombogenicity = [(C14:0 + C16:0 + C18:0)/[(0,5 x ΣMUFA) + (0,5 x Σω6 + (3 x Σω3) + (Σω3/Σω6)];
- Hypercholesterolemic = C12:0 + C14:0 + C14:1+ C16:0 + C16:1;
- Hypocholesterolmic = C18:1 cis9 + C18:2 ω6 + 20:4ω6 + C22:5ω3
- h:H = (C18:1 cis9 + C18:2 ω6 + 20:4ω6 + C22:5ω3)/(C14:0 + C16:0).

### 2.5 Statistical analysis

The results were evaluated using the single-characteristic model: Yij = μ + Ti + eij.

Where Yij = observed value of the dependent variable; μ = overall mean; Ti = effect of treatment i (i = 1 to 5); and eij = experimental error. An analysis of variance (ANOVA) and a regression set at 5% probability were used to analyze the results. The variables Chroma and hue angles did not show normal distribution, they were evaluated using the Kruskal-Wallis test. In all cases, using PROC GLIMMIX from the Statistical Analysis System–SAS version 9.1 (SAS, 2009).

## 3. Results

### 3.1 Chemical composition, physical quality and pH of meat

Meat from lambs fed the Tuc diet showed the highest amount of lipids (4.78g/kg), followed by the Cup (4.72) and Control (4.69) diets, which showed no difference between them, and the Palm diet the lowest value was observed (3.82g/kg) (P = 0.02) (Table 3). Protein was higher (g/kg) in animals on the Cup (22.39) and Tuc (23.29) diets, the intermediate value occurred on the Palm diet (21.41) and the lowest value was found on the control diet (20.19) (P < 0.01). Diets did not influence DM and ashes composition (P > 0.05).

**Table 3:** Chemical composition (%) of meat from lambs fed with different oilseed meal.

Item	Diet				SEM^2^	*P*-value
	Control	Cup	Palm	Tuc		
Dry matter	27.68	26.95	27.10	28.28	0.21	0.44
Ashes	1.20	1.06	1.12	1.13	0.01	0.19
Total lipids	4.69^ab^	4.72^ab^	3.82^b^	4.78^a^	0.11	0.02
Protein	20.19^b^	22.39^a^	21.41^ab^	23.29^a^	0.65	<0.01

Diets: Control = No addition of Amazon oilseed cakes; Cup = inclusion of cupuassu cake; Palm = inclusion of palmiste cake; Tuc = inclusion of tucuma cake. In the same row means with different superscripts differ significantly (*P*<0.05).

^1^Variables analyzed by the Test Kruskal.

^2^SEM = standard error of the mean.

The force (N/cm^3^) used for shear was higher in meat from animals on the Cup diet (27.85), followed by Palm (25.98) and Tuc (26.3), which did not differ from each other, and the lowest value observed in the meat of animals fed the control diet (19.90) (P = 0.02) (Table 4). The diets did not influence the water-holding capacity (%) (P > 0.05), however, there was a difference in cooking losses (%), being higher in the control diet (35.78), followed by Cup (33.30), Tuc (29.70) and Palm (27.95) (P < 0.01).

**Table 4:** Physical quality parameters and pH of meat from lambs fed with different oilseed meal.

Item	Diet				SEM^1^	*P*-value
	Control	Cup	Palm	Tuc		
pH	5.76	5.75	5.72	5.79	0.03	0.91
Shear force (N/cm^3^)	19.90^b^	27.85^a^	25.98^ab^	26.30^ab^	0.08	0.02
WHC (%)	26.97	30.65	29.51	26.27	0.60	0.54
Cooking losses %	35.78^a^	33.30^ab^	27.95^c^	29.70^bc^	0.54	<0.01
L*	37.78^c^	36.74^c^	46.26^a^	42.08^b^	0.45	<0.01
a*	19.17^b^	17.81^b^	22.73^a^	18.16^b^	0.38	<0.01
b*	9.90^b^	10.00^b^	11.77^a^	8.91^b^	0.34	<0.01
Chroma*^2^	45.64^b^	37.95^b^	71.43^a^	38.95^b^	-	<0.01
Hue Angle^2^	42.81^b^	72.43^a^	46.81^b^	31.93^b^	-	<0.01

Diets: Control = No addition of Amazon oilseed cakes; Cup = inclusion of cupuassu cake; Palm = inclusion of palmiste cake; Tuc = inclusion of tucuma cake. WHC = Water-holding capacity; In the same row means with different superscripts differ significantly (P<0.05).

^1^SEM = standard error of the mean; ^2^Variables analyzed by the Test Kruskal.

The colors (L*, a* and b*) were also influenced by the inclusion of co-products in the diets (P < 0.01): Color L* was more lighter in the Palm diet (46.26), followed by Tuc (42.08), Control (37.78) and Cup (36.74) that did not differ (Table 4). Color a* was more intense in the Palm treatment (22.73), the intermediate value observed in Control (19.17), Cup (17.81) and Tuc (18.16) did not differ. In color b*, the intensity was higher in Palm lambs (11.77), followed by Cup (10.0), Control (9.9) and Tuc (8.91), which were equal to each other. The Chroma color was more intense in Palm (71.43), equal and less intense in Control (45.64), Cup (37.95) and Tuc (38.95). The matrix angle was higher in the Cup diet (72.43) and the same in the other diets (Control– 42.81; Palm– 46.81; Tuc– 31.93).

### 3.2 Total fatty acids and profile in *Longissimus lumborum* muscle

The total of FA content (mg/g of meat) was not affected by the diets and presented an average of 212 mg (Table 5). Thirty-eight FA and 3 dimethylacetals (DMA) were identified in the samples, but only 8 FA (14:0, 16:0, 16:1c9, 18:0, 18:1c9, 18:1c11, 18:2n-6 and 20:4n-6) in an amount greater than 10 mg/g of total FA. Others include iso and ante-iso-branched chain FA (BCFA, i-15:0, a-15:0, i-16:0, i-17:0 and a-17:0), isomers derived from biohydrogenation of rumen (trans and cis-18:1 isomers, CLA), long chain PUFA (20:2n-6, 20:3n-6, 20:5n-3, 22:4n-6, 22:5n-5 and 22: 6n-3), Saturated FA (10:0, 12:0, 15:0, 17:0, 20:0 and 22:0) and others.

**Table 5:** Total fatty acids (FA) (mg∕100g of meat) of *longissimus lumborum* muscle from lambs fed with different oilseed meal.

FA profile	Diets				SEM^1^	*P*-value
	Control	Cup	Palm	Tuc		
10:0	0.98	1.03	0.92	1.09	0.01	0.46
12:0	0.53^b^	0.65^b^	0.97^a^	1.16^a^	0.09	<0.01
14:0	16.2^b^	16.7^b^	20.6^a^	21.5^a^	0.10	<0.01
i-15:0	0.52^b^	0.73^b^	0.64^b^	1.08^a^	0.09	<0.01
a-15:0	0.66^b^	0.95^ba^	0.72^b^	1.03^a^	0.10	0.05
14:1*c*9	0.53	0.49	0.72	0.75	0.10	0.21
15:0	1.80	2.03	2.14	2.40	0.17	0.12
i-16:0	0.80^b^	1.19^a^	0.92^ba^	1.26^a^	0.12	0.04
16:0	231	220	237	221	5.30	0.10
i-17:0	2.01	2.26	2.50	2.36	0.16	0.19
16:1*c*7	2.14^b^	2.30^b^	2.30^b^	2.64^a^	0.08	<0.01
16:1*c*9	14.9	13.2	15.7	13.4	1.33	0.52
a-17:0	2.80	3.32	2.91	3.00	0.23	0.44
17:0	7.38	6.54	6.78	7.56	0.53	0.48
17:1*c*9	4.78	3.51	3.97	3.73	0.37	0.12
18:0	156^b^	188^a^	176^ba^	203^a^	10.0	0.03
18:1*t*6/*t*7/*t*8	1.95	2.31	2.01	2.42	0.15	0.09
18:1*t*9	2.24	2.22	1.95	2.08	0.13	0.40
18:1*t*10	5.06	5.17	4.46	4.36	0.79	0.84
18:1*t*11	5.54	6.40	5.55	6.70	0.63	0.46
18:1*c*9	468^a^	429^b^	429^b^	411^b^	8.50	<0.01
18:1*c*11	10.47	8.23	9.53	9.08	0.58	0.08
18:1*c*12	1.08	9.72	1.02	1.23	0.15	0.67
18:1*c*13	1.00^a^	0.42^c^	0.66^bc^	0.85^ba^	0.10	<0.01
18:1*t*16/*c*14	0.77^b^	0.88^ba^	1.03^ba^	1.15^a^	0.09	0.05
18:2n-6	24.2	35.9	26.3	30.0	4.41	0.29
20:0	0.76^c^	3.52^a^	0.95^c^	2.13^b^	0.29	<0.01
18:3n-3	1.86	2.34	2.19	2.57	0.20	0.13
18:2*c*9*t*11^2^ (CLA)	3.53	3.33	2.79	2.93	0.22	0.09
20:2n-6	0.18	0.21	0.15	0.17	0.03	0.60
18:3c9*t*11*c*15/20:3n-9	2.45	2.62	3.21	2.84	0.49	0.73
22:0	0.17^b^	0.44^a^	0.24^b^	0.30^ba^	0.06	0.04
20:3n-6	0.80	1.08	0.92	0.94	0.17	0.72
20:4n-6	10.3	13.0	12.3	12.8	2.52	0.86
20:5n-3	0.67	0.63	0.95	0.95	0.16	0.38
22:4n-6	1.13	1.03	1.13	1.01	0.22	0.96
22:5n-3	1.26	1.45	1.86	1.93	0.27	0.28
22:6n-3	0.24	0.24	0.35	0.50	0.09	0.15

Diets: Control = No addition of Amazon oilseed cakes; Cup = inclusion of cupuassu cake; Palm = inclusion of palmiste cake; Tuc = inclusion of tucuma cake.

^1^ SEM. standard error of the mean; ^2^The 18:2c9t11 might include the 18:2t7c9 and 18:2t8c10 as minor isomers. In the same row means with different superscripts differ significantly (*P*<0.05).

Only 3 of the top 8 FA were affected by diets (mg/g of total FA). Oleic acid (18:1c9) was the main one in all treatments, with a maximum value in the Control (468.0) and similar in the other diets, with an average of 423 mg/g (P < 0.01). Stearic acid (18:0) was lowest in Control (156 mg/g total FA), highest with Cup (188) and Tuc (203), and intermediate with Palm (176) (P = 0.03). Myristic acid (14:0) was more abundant with Palm and Tuc (mean 21.1), control and Cup had a mean of 16.5 mg/g (P < 0.01).

Eight of the minor FAs were affected by diets (P < 0.05). Lauric acid (12:0) follows the same pattern described for 14:0. The Tuc diet increased i-15:0 and 16:1c7 in relation to the other treatments and i-16:0 was lower with the Control. The FAs 20:0 and 22:0 were higher with Cup and lower with Control and Palm. The 18:1t16/c14 was higher with Tuc and lower with Control (P = 0.05). Finally, the 18:1c13 was higher with Control (1.0) and lower with Cup (0.42) (P < 0.01). None of the PUFAs (18:1t10, 18:1t11 and 18:2c9, t11), and biohydrogenation derivatives, were affected by diets (P > 0.05).

Sums of SFA, trans-MUFA, n-3 and n-6 PUFA were unaffected by diets (P > 0.05). However, the cis-MUFA sum was higher with the Control (503 mg/100 g of meat), as described for 18:1c9 (P < 0.01). The sum of BCFA (mg/100 g of meat) was lowest with Control (6.80) and highest with Cup (8.46) and Tuc (8.74) (P = 0.03) (Table 6).

**Table 6:** Fatty acids profile (mg/100 g of meat) of *Longissimus lumborum* muscle from lambs with different oilseed meal.

Item	Diet				SEM^1^	*P*-value
	Control	Cup	Palm	Tuc		
**DMA profile**						
DMA-16:0	6.65	6.08	8.41	6.89	1.5	0.72
DMA-18:0	4.89	8.57	7.38	7.35	1.4	0.30
DMA-18:1	0.98	1.23	1.14	1.01	0.3	0.90
**Sums**						
SFA	415	439	445	460	12.9	0.14
BCFA	6.80^b^	8.46^a^	7.70^ba^	8.74^a^	0.5	0.03
*cis*-MUFA	503^a^	458^b^	463^b^	443^b^	9.5	<0.01
*trans*-MUFA	15.6	17.0	15.0	16.7	1.2	0.63
PUFA	40.7	55.8	46.2	50.8	8.1	0.60
n-3 PUFA	4.06	4.66	5.35	5.94	0.7	0.23
n-6 PUFA	36.6	51.2	40.9	44.9	7.2	0.53
**SCD activity indices** ^**2**^						
SCDi16	6.03	5.67	6.23	5.71	0.5	0.86
SCDi17	39.0	34.8	36.7	33.3	1.7	0.14
SCDi18	75.0^a^	69.6^b^	71.0^ba^	67.1^b^	1.4	<0.01

Diets: Control = No addition of Amazon oilseed cakes; Cup = inclusion of cupuassu cake; Palm = inclusion of palmiste cake; Tuc = inclusion of tucuma cake.

1 Standard error of the mean

^2^Desaturase stearoyl-CoA (SCD) activity indices were calculated as follow: SCDi16 = (16:1/(16:1+16:0)

*100). SCDi17 = (17:1/(17:1+17:0)*100). SCDi18 = (18:1/(18:1+18:0)*100. In the same row means with different superscripts differ significantly (P<0.05).

The most nutritionally relevant FAs and the sum of FAs expressed in mg/100 g of fresh meat were different for 18:2 n-6 (P = 0.04) and n-6 PUFA (P = 0.05). The observed values were higher in the Cup diet in relation to the Palm.

Differences reported for 18:0, 18:1c9 and cis-MUFA expressed in mg/g of total FA do not appear when expressed in mg/100 g of meat (P > 0.05). These values accumulate the random variability associated with the FA profile and the internal standard quantification method and, therefore, show greater variance than when expressed as a proportion of the total FA. This certainly explains why the differences in 18:1c9 or cis-MUFA between the Control and the other treatments, expressed in mg/100g of meat, did not show significance.

### 3.3 Sums and proportions of the main fatty acids

Diets influenced only 18:2n-6 (P = 0.04) and n-6 PUFA (P = 0.05) (Table 7). The Cup diet scored the highest (175) and the Palm diet the lowest (127) for 18:2n-6. As for n-6 PUFA, Palm obtained the lowest values (193) and Cup the highest values (248).

**Table 7:** Sums and proportions (mg/100 g of meat) of the main fatty acids in the *Longissimus lumborum* muscle of lambs fed with different oilseed meal.

Items	Diets				SEM^1^	*P-value*
	Control	Cup	Palm	Tuc		
16:0	1550	1206	1324	1196	235	0.69
18:0	1083	1020	993	1128	202	0.96
18:1c9	3074	2352	2344	2228	401	0.44
18:2n-6	147^ab^	175^a^	127^b^	148^ab^	10.7	0.04
18:3n-3	12	13	11	13	1.5	0.77
18:2c9t11	24	18	15	16	2.7	0.13
20:4n-6	57	61	56	61	4.1	0.65
SFA	2829	2399	2507	2530	467	0.93
*cis-MUFA*	3306	2518	2529	2404	426	0.44
*trans-MUFA*	106	90	81	91	15.7	0.73
PUFA	278	304	248	281	14.9	0.10
n-3 PUFA	25	25	25	30	2.7	0.40
n-6 PUFA	216	248^a^	193^b^	220	12.6	0.05
Total FA	6634	5433	5483	5429	912	0.74

Diets: Control = No addition of Amazon oilseed cakes; Cup = inclusion of cupuassu cake; Palm = inclusion of palmiste cake; Tuc = inclusion of tucuma cake. In the same row means with different superscripts differ significantly (*P*<0.05).

^1^ SEM, standard error of the mean.

### 3.4 Lipid fraction indices

With the exception of the “h” index (P = 0.01), the lipid fraction indices were not significant (P > 0.05) (Table 8). Hypocholesterolemics were higher on the control diet (50.4), followed by Cup (47.9), Palm (47.0) and Tuc (45.6), respectively. However, the TI differed between the control diet (4.77) and Tuc (6.18); and Cup (25.40) differed from Palm (27.70) in the hypercholesterol index, with Palm’s value being higher.

**Table 8:** Indices of the lipid fraction of fatty acids of the *Longissimus lumborum* muscle (mg∕ 100g of meat) of lambs fed with different oilseed meal.

Items	Diet				SEM^1^	*P*-value
	Control	Cup	Palm	Tuc		
IA^2^	0.53	0.54	0.61	0.60	0.02	0.06
IT^3^	4.77^a^	5.92	5.65	6.18^b^	0.48	0.22
h^4^	50.4^b^	47.9^ab^	47.0^a^	45.6^a^	0.93	0.01
H^5^	26.6	25.4	27.7	26.1	0.59	0.06
h:H index	1.91	1.9	1.7	1.75	0.06	0.07

Diets: Control = No addition of Amazon oilseed cakes; Cup = inclusion of cupuassu cake; Palm = inclusion of palmiste cake; Tuc = inclusion of tucuma cake. In the same row means with different superscripts differ significantly (P<0.05).

^1^ Standard error of the mean

^2^Atherogenicity index

^3^Thrombogenicity Index

^4^Hypocholesterolemic

^5^Hypercholesterolemic.

## 4. Discussion

### 4.1 Chemical composition, physical quality and pH of meat

Total lipid of meat is one of the main determinants of sensory and instrumental meat quality characteristics [30,31]. In the present study, the average intramuscular fat was 4.5 g/100g, which is within the range commonly found for Dorper × Santa Inês lambs [32]. Intramuscular fat only significantly decreased in the Palm treatment, which may be related to its high 12:0 content and its supposed anti-adipogenic effects [16]. However, the Tuc treatment was also 12:0 rich, but the intramuscular fat, although numerically lower than the control, did not differ significantly.

Despite diets formulated to be isoproteic, there was a difference between treatments. This fact possibly influenced the availability in the diet and, consequently, the protein composition of the meat.

In meat, water retention capacity is a very important quality attribute, as it has a direct influence on yield, visual acceptability and sensory characteristics at the time of consumption [33,34]. Several pre- and post-mortem factors influence the water retention capacity of meat, including the animal’s diet [35]. However, the use of co-products did not influence this characteristic.

Consumers associate meat color as an indicator of quality and freshness at the time of choice [36]. The instrumental evaluation of color detected that meat from the Palm treatment differed from the others, presenting the highest L*, a*, b* and chroma* (P < 0.05). The reasons for this are not clear, but may be linked to the type of phenolic compound present in the palm kernel cake, since phenolic compounds have an influence on the color, as they have a specific effect against myoglobin oxidation and meat discoloration [37,38]. Saeed [39] also observed an increase in L* in *Longissimus lumborum* when associating palm oil cake with corn. A decrease in this variable has been associated with browning of the meat, due to the transformation of myoglobin into metmyoglobin, which is less desirable for the consumer [40].

Lamb meat cooking losses are correlated with intramuscular fat and carcass fat [41,42]. Our data are consistent with this, as the lowest cooking losses were reported for palm meat, followed to a lesser extent by Tuc meat.

Shear strength and meat tenderness are also frequently associated with intramuscular fat [43,44]. In the present work, the opposite was observed, with the highest shear strength verified in the Cup and Tuc treatments, where the highest values of total lipids were also observed (Table 3). Despite this, it always remained below 27.85 N/cm^2^, thus, the treatments resulted in meats classified as tender or of medium tenderness [45,46].

### 4.2 Total fatty acids and profile in *Longissimus lumborum* muscle

The most consistent effects of diets on the FA composition of *Longissimus lumborum* meat were at 12:0, 14:0, 18:0, 18:1c9, 20:0 and 22:0 and are, in general, partially explained by differences in FA composition of diets. The Palm and Tuc diets (rich in 12:0 and 14:0) increased FA ratios in muscle. The comparatively low incorporation of 12:0 into the tissues of animals fed 12:0-rich fat sources has been reported in lambs [16] rabbits [47] and pigs [48]. The explanation for this is possibly related to the more direct absorption pathways of 12:0, which can be more easily transported as non-esterified FA to the liver (portal vein), without the need to be reassembled into triacylglycerols and, subsequently, incorporated into chylomicrons, to proceed via lymphatic transport compared to longer-chain FAs [49]. Furthermore, 12:0 may undergo more extensive post-absorptive oxidation than other long-chain FAs, already observed in humans and largely extended to 14:0 [47].

Another consistent effect of the diets on tissue FA is the increase of 18:0, 20:0 and 22:0 (mg/g of total FA) in the muscle of animals on the Cup diet. Cupuassu seed, along with cocoa butter and mango seed oil, are quite unusual among vegetable fat sources as they contain large amounts of 18:0 [50]. In ruminants, 18:0 is often the main absorbed FA, as it is the main biohydrogenation product of C18 unsaturated FA in the rumen [51], being extensively desaturated in tissues to 18:1c9 by SCD [52]. The Cup diet provided a large ratio of 18:0 and 18:1c9 and the highest proportion of C18 FA (about 86%, compared to 75% in the control, 49% in Palm and 31% of total FA in Tuc), therefore a large embedding of 18:1c9 can be expected. However, the 18:0 was the only big C18 FA that consistently rose in the meat of the Cup treatment.

The effects of 12:0 FA and 12:0-rich fat sources on the ruminal microbiota are large and may include defaunation, inhibition of methanogenesis and fibrolytic activity [53]. Another indication of the potentially destabilizing effect of 12:0 in the rumen is the induction of the trans-10 displaced biohydrogenation pathway as reported by [16]. Normal rumen biohydrogenation pathways generate 18:1t11 as the main intermediate [51]. However, when ruminants are fed diets that favor low ruminal pH, it is frequent to observe a change in biohydrogenation pathways where 18:1t10 becomes the main biohydrogenation intermediate (trans-10 shift) and, therefore, becomes if the main trans-18: 1 isomer deposited in tissues [27]. In the present work, no change in rumen biohydrogenation pathways was apparent for any diet, as the trans-18:1 ratio remained low (below 17 mg/g total FA) and 18:1t10 remained less than 18:1t11. Parente [16] reported that dietary Babassu oil (with 49% of 12:0) induced the occurrence of the trans-10 shift, but the diets contained a lower roughage/concentrate ratio (3:7 vs. 4:6) and the dose of 12:0 was much higher, as they supplemented the diets with 40 g/kg of Babassu oil.

### 4.3 Sums and proportions of the main fatty acids

Edible fats from ruminants are the main dietary source of CLA for humans. Some CLA isomers, such as rumenic acid (18:2c9,t11), have health-promoting actions, notably anticancer [54]. Thus, enrichment of ruminant meat with CLA is often a goal in experiments with growing ruminants [27,55]. Consistent with the low 18:1 trans content, the 18:2c9,t11 content was also relatively low (about 3.5 mg/g total FA in the Control) and tended to decrease with the inclusion of the oilseed cakes studied. All have lower PUFA content than the control.

Furthermore, the nut cake diets decreased the 18:1c9 ratio in meat. 18:1c9 is considered a desirable FA, particularly when compared to SFA and trans FA [56,57].

The BCFA in the meat were increased with the oilseed cake, mainly from the Cup and Tuc diets, when compared to the control, due to the increase in iso-15:0, anteiso-15:0 and iso-16:0, which are BCFA of microbial origin. Indeed, microorganisms in the rumen synthesize iso and anteiso BCFA from dietary branched-chain amino acids, which are structural lipid constituents of bacterial membranes. Bacterial cells can escape from the rumen and be later absorbed and consequently incorporated into animal tissues. Interest in this class of compounds has increased due to their biological effects and potential pro-health benefits [58].

### 4.4 Lipid fraction indices

Some FAs have a pharmacological effect and are associated with the prevention of cardiovascular diseases, such as atherosclerosis and thrombosis [59–61]. Studies demonstrate the importance of conscious consumption of these compounds and, for example, their beneficial role in modulating the function of the endothelium [61]. Thus, some indices were created to help group FAs according to their effects on the body. Atherogenicity (AI) and thrombogenicity (TI) indices quantify the potential to stimulate platelet aggregation, the possibility of atherosclerosis, thrombosis and stroke [62,63]. The higher the AI and TI value, the lower the amount of antiatherogenic and antithrombogenic fatty acids and the greater the potential for coronary disease [64].

The smaller values of AI present a greater protective potential for coronary artery diasese. however, the AI was below the recommended value meat (1.0) [65], but they were better than the average found [66], who observed an average value of 0.56.

The highest value of h in the control treatment was possibly due to the FA composition of the diet, surpassing those found in diets with the addition of co-products. However, these were higher values than those observed by Nascimento [67] (11.75 to 14.74), which included corn co-products in sheep diets.

The h:H ratio the bigger the better. The average found (1.9) is acceptable and poses no risk to human health [7,68,69]; They are within the range observed for meat (1.27 to 2.79) [70].

## 5. Conclusion

The inclusion of oilseed cakes influences the chemical composition, physical parameters, composition and fatty acid profile of the meat. Despite the increase in the thrombogenicity index in the tucuma diet and the shear force with the inclusion of co-products, there is a reduction in cooking losses and does not influence the indicators of atherogenicity and cholesterolemia. We recommend further studies for possible inclusion in lamb diets.
